# Components of Adenovirus Genome Packaging

**DOI:** 10.3389/fmicb.2016.01503

**Published:** 2016-09-23

**Authors:** Yadvinder S. Ahi, Suresh K. Mittal

**Affiliations:** ^1^Department of Comparative Pathobiology, Purdue UniversityWest Lafayette, IN, USA; ^2^Purdue University Center for Cancer Research, Purdue UniversityWest Lafayette, IN, USA; ^3^Purdue Institute for Immunology, Inflammation and Infectious Diseases, Purdue UniversityWest Lafayette, IN, USA

**Keywords:** adenovirus, genome packaging, IVa2, L4 33K, L4 22K, ATPase, packaging domain, portal vertex

## Abstract

Adenoviruses (AdVs) are icosahedral viruses with double-stranded DNA (dsDNA) genomes. Genome packaging in AdV is thought to be similar to that seen in dsDNA containing icosahedral bacteriophages and herpesviruses. Specific recognition of the AdV genome is mediated by a packaging domain located close to the left end of the viral genome and is mediated by the viral packaging machinery. Our understanding of the role of various components of the viral packaging machinery in AdV genome packaging has greatly advanced in recent years. Characterization of empty capsids assembled in the absence of one or more components involved in packaging, identification of the unique vertex, and demonstration of the role of IVa2, the putative packaging ATPase, in genome packaging have provided compelling evidence that AdVs follow a sequential assembly pathway. This review provides a detailed discussion on the functions of the various viral and cellular factors involved in AdV genome packaging. We conclude by briefly discussing the roles of the empty capsids, assembly intermediates, scaffolding proteins, portal vertex and DNA encapsidating enzymes in AdV assembly and packaging.

## Introduction

Adenoviruses (AdVs) are icosahedral nonenveloped DNA viruses belonging to the family *Adenoviridae* and are ~90 nm in diameter. They are known to infect a wide range of species. Some of the AdVs can cause life threatening disease in immunocompromised hosts. Human AdVs (HAdVs) constitute more than 70 types (Hage et al., 2015), which are sub-classified into species A through G based on genome sequence, agglutination properties, immune cross-reactivity and genome organization. They are known to cause respiratory infections, conjunctivitis and gastroenteritis in normal individuals (Russell, 2009). The HAdVs belonging to species B and C are well characterized among HAdVs.

In the last three decades, research on AdVs has focused extensively on their development as gene delivery vectors. Understanding of the AdV assembly process is important for the development of improved drug delivery vehicles and vectors for gene delivery, for studying the assembly and packaging of other animal viruses or macromolecular complexes, and for better understanding of virus evolution. Additionally, the AdV assembly pathway can lead to the discovery of novel anti-viral targets and nanotechnological tools.

**Figure 1:**
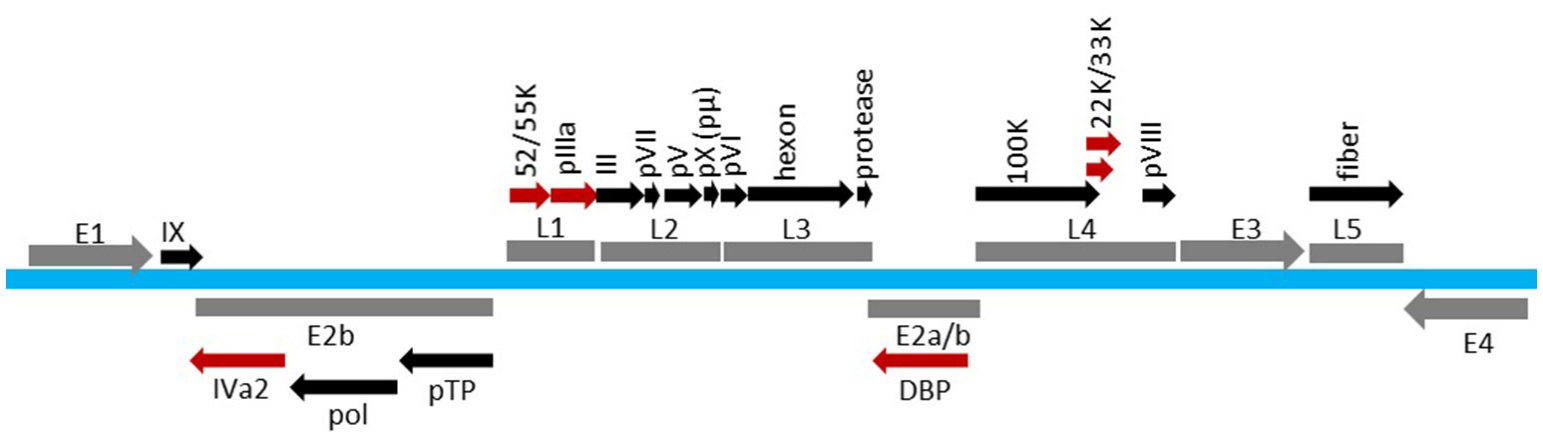
**Transcription map of HAdV-C5 genome**. Viral genome is shown in blue. The early regions (E1, E2, E3, and E4) and the late regions (L1, L2, L3, L4, and L5) are shown in gray. Proteins expressed from various regions are indicated above or below the region. Factors involved in AdV packaging are shown in red. The map is not drawn to scale.

AdV has a pseudo *T* = 25 icosahedral capsid enclosing a linear dsDNA genome varying in size (26–45 Kbp) depending on the AdV serotype. The HAdV serotype 5 (HAdV-C5) capsid is composed of a total of 14 proteins (Table 1; Reddy and Nemerow, 2014). The hexon (protein II of 109 kDa in size), penton base (protein III of 63.3 kDa in size) and fiber (protein IV of 61.9 kDa in size) are referred to as the major capsid proteins. The 240 trimers of hexon represent the most abundant capsid component. Pentons occupy the 12 vertices of the icosahedron. Each penton is composed of a trimeric penton base with a protruding trimeric fiber. The minor capsid components, protein IIIa (63.5 kDa), VIII (15.4 kDa), and IX (14.4 kDa), function primarily as cement proteins by connecting the major structural units with each other and the viral core (Vellinga et al., 2005; Russell, 2009). Protein VI, in addition to serving as a cement protein, also plays important roles in virion maturation, entry, trafficking and early gene expression (Wiethoff et al., 2005; Wodrich et al., 2010; Moyer et al., 2011; Graziano et al., 2013; Schreiner and Wodrich, 2013; Luisoni et al., 2015). The viral core contains the dsDNA genome (35,938 Kbp in HAdV-C5) associated with four proteins: V (41.6 kDa), VII (19.4 kDa), μ (4 kDa), and terminal protein (TP) [55 kDa], in addition to the viral protease (23 kDa) (Everitt et al., 1973; Rux and Burnett, 2004; Vellinga et al., 2005; Perez-Berna et al., 2015). IVa2, L4 33K, L4 22K, E2 72K, and L1 52/55K proteins are involved in capsid assembly and/or genome packaging (Nicolas et al., 1983; Roovers et al., 1990; Christensen et al., 2008; Wohl and Hearing, 2008; Wu et al., 2012, 2013; Perez-Berna et al., 2014; Ahi et al., 2015; Condezo et al., 2015). The viral proteins with a role in genome packaging are depicted in the transcription map of HAdV-C5 (Figure 1).

**Table 1:** **Protein composition of AdV empty and mature capsids**.

**AdV protein**	**Presence in empty or mature capsids**	**Copy number of monomer**	**Location**	**Function/s**	**References**
II (Hexon)	Both	720	Capsid (exterior)	Structure	Liu et al., 2010; Reddy et al., 2010; Benevento et al., 2014; Reddy and Nemerow, 2014
III (Penton base)	Both	60	Vertex (exterior)	Structure, Entry	Liu et al., 2010; Reddy et al., 2010; Benevento et al., 2014
IIIa	Both	68 ± 2	Capsid (exterior)? Vertex (Interior)?	Cement	Liu et al., 2010; Reddy et al., 2010; Benevento et al., 2014; Reddy and Nemerow, 2014
Fiber	Both	36	Vertex (exterior)	Structure, Entry	Liu et al., 2010; Reddy et al., 2010; Benevento et al., 2014
V	Mature	157	Core	Core condensation	Takahashi et al., 2006; Liu et al., 2010; Reddy et al., 2010; Benevento et al., 2014; Perez-Berna et al., 2015
VI	Both	342 ± 4	Vertex (Interior)?	Cement, Entry, Trafficking, early gene expression	Wiethoff et al., 2005; Liu et al., 2010; Reddy et al., 2010; Wodrich et al., 2010; Moyer et al., 2011; Graziano et al., 2013; Schreiner and Wodrich, 2013; Benevento et al., 2014; Luisoni et al., 2015
VII	Mature	527 ± 44	Core	Core condensation	Liu et al., 2010; Reddy et al., 2010; Benevento et al., 2014; Perez-Berna et al., 2015
VIII	Both	128 ± 3	Capsid (interior)	Cement	Liu et al., 2010; Reddy et al., 2010; Benevento et al., 2014
IX	Both	247 ± 2	Capsid (exterior)	Cement	Liu et al., 2010; Reddy et al., 2010; Benevento et al., 2014
TP	Both	2	Core	DNA replication	Liu et al., 2010; Reddy et al., 2010; Benevento et al., 2014
mu	Mature	290 ± 18	Core	Core condensation	Liu et al., 2010; Reddy et al., 2010; Benevento et al., 2014; Perez-Berna et al., 2015
AVP	Both	15 ± 5	Core	Maturation	Rancourt et al., 1995; Liu et al., 2010; Reddy et al., 2010; Benevento et al., 2014; Perez-Berna et al., 2014, 2015; Condezo et al., 2015
IVa2	Both	7 ± 1	Unique vertex	Packaging	Christensen et al., 2008; Ostapchuk and Hearing, 2008; Ahi et al., 2013
L4 33K	Empty	?	Unique vertex	Packaging	Christensen et al., 2008; Ostapchuk and Hearing, 2008; Ahi et al., 2013, 2015; Wu et al., 2013
L4 22K	Empty	?	Unique vertex (?)	Packaging	Ostapchuk et al., 2006; Wu et al., 2012; Guimet and Hearing, 2013
L1 52/55K	Empty	50	Capsid (interior)	Packaging, Genome retaining?	Perez-Berna et al., 2014
72K (DBP)	Both	?	Unique vertex	Capsid assembly, DNA replication	Nicolas et al., 1983; Ahi et al., 2013

*Copy number of monomer, location, and function of each protein are indicated. The question mark sign (?) in the copy number column indicates that the information is unknown. The question mark (?) in the location and function column indicates the predicted location and function*.

**Figure 2:**
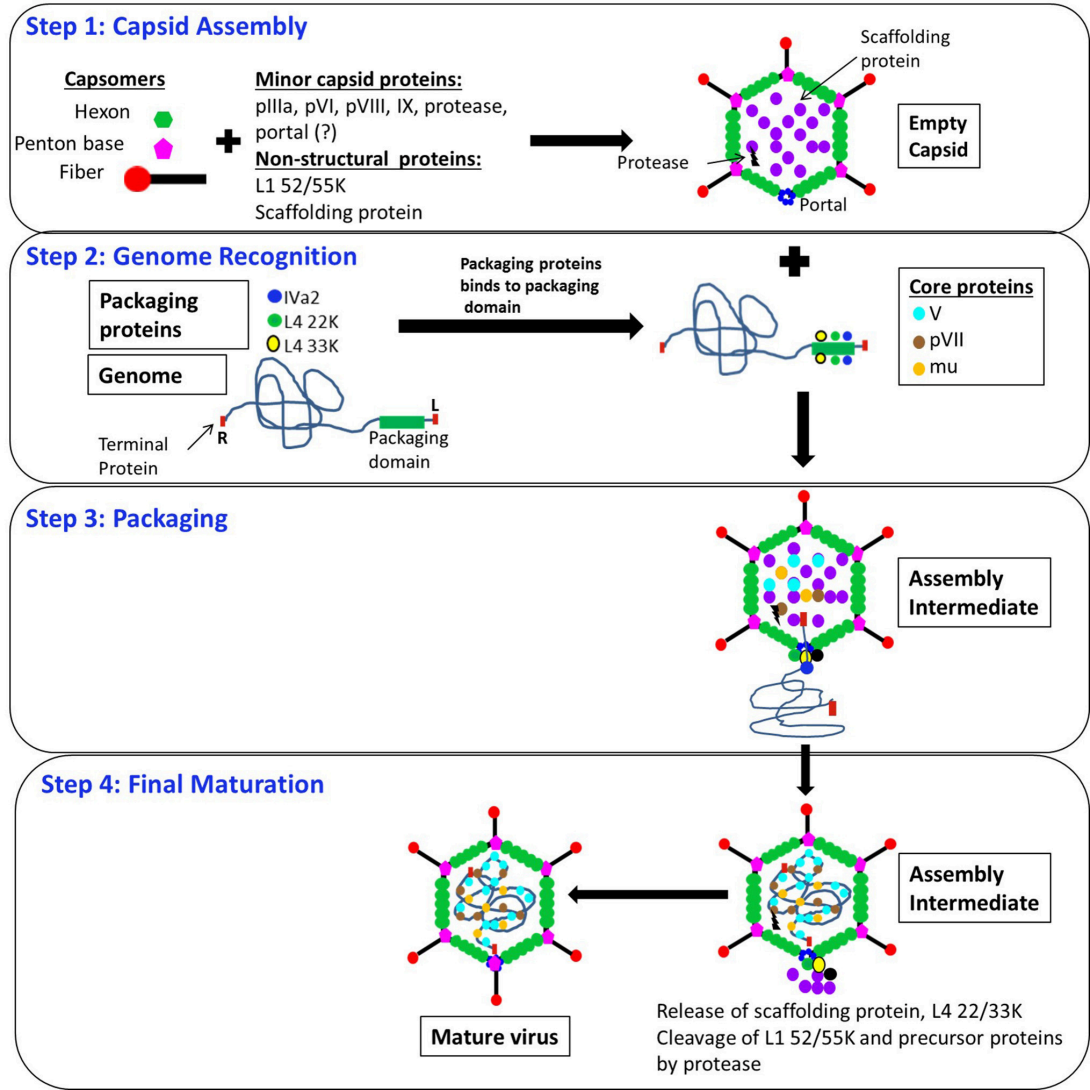
**Predicted model of AdV assembly and packaging. Step 1:** Capsid assembly: Empty capsids are assembled from hexon trimers and pentons, along with the minor capsid proteins (pIIIa, pVI, pVIII, IX, protease and portal) and the non-structural proteins (L1 52/55K and the scaffolding protein). **Step 2**: Genome recognition: Packaging proteins IVa2, L4 33K, L1 52/55K, and L4 22K bind to the packaging domain located close to the left end of the genome. The genome is associated with pTP at each end. **Step 3**: Packaging: The genome is inserted into the empty capsids through an unknown portal located at the unique vertex by the action of IVa2, L4 22K, and L4 33K. The core proteins V, VII and mu are incorporated into the empty capsids during or after genome incorporation. **Step 4**: Final maturation: Scaffolding protein (not known), L4 22K and 33K are released during or after genome incorporation. The virus encoded protease cleaves its substrates pIIIa, L1 52/55K, pVI, pVII, pVIII, mu and pTP, resulting in conformational changes in the capsid structure and maturation of the virus particle. pVI, precursor VI; pVII, precursor VII; pVIII, precursor VIII; pTP, precursor terminal protein.?, indicates that the identity is not known. L and R indicate left and right ends of the viral genome, respectively.

Viruses may follow one of the two possible assembly pathways (Casjens, 1997). (1) Concomitant assembly: the capsids are assembled by sequential addition of the capsomers around the viral genome. This kind of assembly is seen in helical viruses, for example the tobacco mosaic virus, in spherical retroviruses and even in some icosahedral viruses, for example SV40 (Kawano et al., 2006). (2) Sequential assembly: The majority of viruses with icosahedral capsids first assemble the empty capsids or procapsids through the interaction of one or more structural units followed by the incorporation or “packaging” of the viral genome into the preformed capsids (Casjens, 1997). AdV assembly is thought to proceed along the sequential pathway that includes the following steps (Figure 2): (1) Assembly of the hexon and penton capsomers followed by the assembly of empty capsids, which also involves the minor capsid proteins and the non-structural proteins; (2) Specific recognition of the viral genome by packaging proteins; (3) Insertion of the viral genome into empty capsids, presumably through a portal located at a unique vertex, and release of scaffolding and some of the packaging proteins; and (4) Cleavage of the precursor proteins by the viral protease, enabling final maturation of viral particles (D'Halluin, 1995; Ostapchuk and Hearing, 2003b, 2005). The assembly of AdV hexon trimers is dependent on a virus-encoded chaperone-like L4 100K protein (Cepko and Sharp, 1982; Hong et al., 2005). In addition to empty and mature virus particles, light and heavy intermediate particles are also detected in AdV infected cells (Edvardsson et al., 1976, 1978; D'Halluin et al., 1978a,b). The light intermediate particles are associated with increasing lengths of DNA derived from the left end of the viral genome (Daniell, 1976; Daniell and Mullenbach, 1978; D'Halluin et al., 1978b), and may represent capsids in the process of packaging (Figure 2, step 3). The heavy intermediate particles possibly represent particles after packaging, but before cleavage of precursor proteins by viral protease (Figure 2, step 4).

## AdV packaging domain

**Figure 3:**
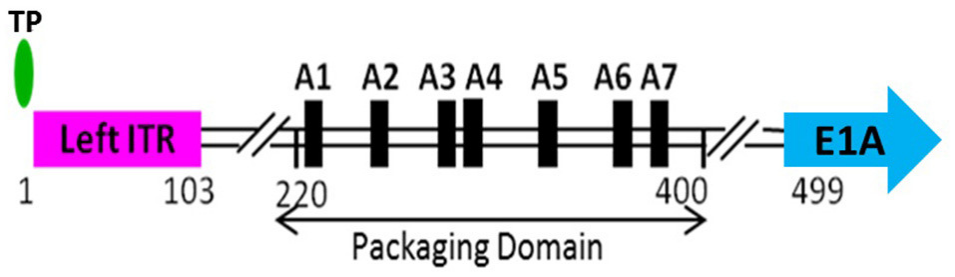
**Schematic depiction of the left end of HAdV-C5 genome**. The packaging domain of HAdV-C5 is located between 220 and 400 bp of the viral genome and consists of seven AT-rich repeats, referred to as A-repeats. The terminal protein (TP) is covalently attached to the 5′-phosphate. The left inverted terminal repeat (left ITR) extends from 1 to 103 bp. Transcription from the early region 1A (E1A) begins at 499 bp.

Selective packaging of the viral genome by capsids poses an interesting puzzle for the completion of the viral life cycle. The viral genome must be recognized selectively by the viral packaging complex for specific incorporation into the capsid. The AdV genome carries a unique sequence, called the packaging domain (PD), comprising a series of AT-rich repeats, located between the left inverted terminal repeat (ITR) and the E1A transcription start site (Hammarskjöld and Winberg, 1980; Kosturko et al., 1982). The PD of HAdV-C5 (Figure 3) is extensively characterized and is located between nucleotides 220 and 400 (Grable and Hearing, 1990, 1992; Tyler et al., 2007). The observation that the incomplete AdV particles are associated with varying lengths of DNA fragments representing the left end of the genome suggested polar packaging of the AdV genome starting from the left end (Daniell, 1976). The location of the HAdV-C5 PD has some flexibility since it can work efficiently if placed close to the right end of the genome as long as it is within 600 bp from the ITR, suggesting that the TP and/or the ITRs may play some role in genome packaging (Hearing et al., 1987). Similar to AdVs, the bacteriophage phi29 and PRD1 genomes contain ITRs and TP covalently linked to the 5′ end of the genomes (Grimes and Anderson, 1989; Savilahti and Bamford, 1993). The TP of phi29 potentiates genome packaging, suggesting a similar mechanism in AdVs. However, a later *in vivo* (refers to cell culture in the context of this review) study showed that the removal of TPs and ITRs did not significantly decrease the efficiency of genome packaging indicating that the AdV genome packaging process may differ from that of phi29 in mechanistic details (Ostapchuk and Hearing, 2003a). Presumably, the distance of the PD from the left end governs the orientation of the genome with respect to the portal. If the location is too far from the left end, it may pose a structural constraint that prevents “threading” of the genome through the portal.

The HAdV-C5 PD consists of seven AT-rich repeats, referred to as A-repeats, numbered A1-A7 from left to right (Ostapchuk and Hearing, 2003a,b). The A-repeats are “functionally redundant,” implying that all A-repeats are not essential for efficient packaging. Some A-repeats are more efficient in directing the genome packaging than others (Hearing et al., 1987; Grable and Hearing, 1990, 1992; Schmid and Hearing, 1997). Specifically, the A1, A2, A5, and A6 repeats are most important and act in pairs of A1 and A2, and A5 and A6 (Schmid and Hearing, 1997). Sequence alignment suggested that they share a bipartite consensus motif 5′-TTTGN_8_CG-3′ and also revealed the presence of a 21 bp spacer between A1 and A2, and between A5 and A6. The A3, A4, and A7 repeats do not show significant homology to the bipartite consensus mentioned above and are not absolutely required for packaging, but may be important for optimal packaging efficiency (Grable and Hearing, 1992; Schmid and Hearing, 1997). The sequence of an 8 nucleotide (nt) spacer between TTTG and CG of the consensus motif is not conserved, but the length is essential for the function of the A-repeats, as increasing the spacing by an additional 4 nt results in significant reduction in packaging efficiency (Schmid and Hearing, 1997). The spacing of 21 bp, which corresponds to two turns of the DNA helix, presumably facilitates the binding of “transacting” factors, recognizing one A-repeat pair on the same face of the helix. Precise nt spacing may also be necessary for essential mutual interactions among the factors that bind to the adjacent A-repeats. The conserved consensus motif may also suggest the binding of the same protein on different A-repeats. Evolution of the AdV PD with redundant A-repeats may be a mechanism to ensure a highly specific recognition of the viral genome by the viral or cellular transacting factors that connect the genome with an empty capsid and set up a packaging initiation complex. Indeed, an AdV genome carrying a single A-repeat has a significant defect in packaging efficiency (Schmid and Hearing, 1998). A synthetic PD consisting of multiple copies of a single A-repeat functions efficiently, demonstrating functional redundancy of A-repeats and establishing A-repeats as a minimal functional unit of the AdV PD. (Schmid and Hearing, 1998).

The presence of transacting factors that bind to the PD was initially suggested by *in vivo* competition experiments. Co-transfection of a plasmid carrying PD sequences and the HAdV genome caused remarkable reduction in final virus yield without showing any significant differences in protein expression and DNA replication (Grable and Hearing, 1992). Similarly, a mutant HAdV-C5 carrying 20 copies of the A6-repeat competed for packaging more effectively than a mutant with 16 copies of the A6-repeat in a co-infection experiment (Schmid and Hearing, 1998). These observations indicated that there is a direct competition among the packaging sequences for essential proteins that bind to the PD and mediate genome packaging. Various studies have used the packaging repeats as probes to discover novel proteins that interact with the PD. The transacting factors that were shown to have a functional association with the PD will be discussed further in the subsequent section.

Alignment of sequences from the left end of AdV serotypes representing different species showed conservation of the bipartite consensus motif of the HAdV-C5 PD, suggesting a common DNA packaging mechanism. There are subtle differences among the A-repeats of PDs of various AdV serotypes. These differences possibly reflect variation in the DNA binding specificities of packaging proteins, but are unlikely to alter the packaging mechanism (Ostapchuk and Hearing, 2003b).

## Proteins involved in AdV DNA packaging

The precise dissection and characterization of AdV PD was the first step toward the understanding of AdV packaging. A logical next step was to identify various proteins that are involved in this complex process. A simple and seemingly straightforward approach was to detect the proteins that interact with the PD sequences; however, the overlap of the E1A transcription enhancer elements with the PD is a confounding factor (Bolwig et al., 1992; Hatfield and Hearing, 1993). Proteins that bind to this region may either act as transcriptional enhancer factors, packaging factors or both. Characterization of the minimal functional unit of the HAdV-C5 PD offered a simple approach that led to the identification of both the viral and cellular proteins that associate with the PD and could play an important role in genome packaging (Table 2). Proteins that associate with the PD include viral proteins: IVa2, L4 33K, L4 22K, L1 52/55K and IIIa, and cellular proteins: chicken ovalbumin upstream promoter transcription factor (COUP-TF), Oct-1 and CCAAT displacement protein (CDP). The role of each of these proteins in AdV packaging is discussed in the sections below.

**Table 2:** **Known AdV packaging factors**.

**Packaging factor**	**Bind to PD**		**Interaction with other factors**	**Role in serotype specificity**	**References**
	***In vivo***	***In vitro***			
IVa2	Yes	Yes	L1 52/55K, L4 33K	No	Zhang and Imperiale, 2000; Tyler et al., 2007
L4 33K	Yes	?	IVa2	?	Ali et al., 2007; Ahi et al., 2013, 2015
L4 22K	Yes	Yes	?	No	Ewing et al., 2007; Wohl and Hearing, 2008; Wu et al., 2012
L1 52/55K	Yes	No	IVa2, IIIa	Yes	Gustin et al., 1996; Perez-Romero et al., 2005; Wohl and Hearing, 2008; Ma and Hearing, 2011
IIIa	Yes	?	L1 52/55K	Yes	Ma and Hearing, 2011

*Question mark indicates “not known”*.

### Viral proteins

Identification of viral proteins involved in AdV packaging relied on DNA-protein electrophoretic mobility shift assay (EMSA) of nuclear extracts of HAdV-C5-infected cells. These EMSA experiments using a probe comprising A1 and A2 repeats of PD, resulted in the identification of two specific complexes: x (larger) and y (smaller) (Zhang and Imperiale, 2000). AdV IVa2 protein, which binds to the downstream element (DE) of the major late promoter, was also identified in complexes x and y. The interaction of IVa2 with the CG motif of A-repeats was essential for the formation of both x and y complexes, whereas the formation of complex y required the binding of IVa2 and an unknown factor of viral origin or virus-induced cellular protein at the TTTG motif of A-repeats (Zhang and Imperiale, 2000). The unknown factor was identified later as the AdV L4 22K protein (Ostapchuk et al., 2006). Ostapchuk et al. provided a more detailed analysis of the infected cell-specific complexes through mutational analysis of the A1 and A2 repeats. Interestingly, instead of two, they reported three complexes, with complexes 1 and 2 being the same as complexes y and x, respectively (Table 3). Introducing mutations in the conserved TTTG and CG motifs of A1 and A2 repeats defined the sequences required for the formation of these complexes. Removal of the CG motif of the A1 repeat inhibited the formation of all three complexes, whereas a mutation in the TTTG motif of the A2 repeat prevented the formation of complex 2. The CG motif of the A2 repeat was shown to be required for the formation of complex 3. These results suggested that AdV IVa2 protein nucleates the formation of a complex on the packaging repeats due to an initial interaction with the CG motif of one of the A-repeats in the PD followed by an interaction with the TTTG motif of a nearby A-repeat. Interaction with the TTTG motif also required the presence of L4 22K protein (Ostapchuk et al., 2006). It is followed by addition of another IVa2 molecule to the complex through an interaction with the CG motif of a second A-repeat. The formation of various complexes *in vitro* correlates with growth properties of the mutant viruses harboring double copies of A1 and A2 repeats mutated at the TTTG and/or CG motif/s. The interaction of IVa2 with the PD *in vivo* was also demonstrated by co-purification of the PD and IVa2 with an anti-IVa2 antibody (Ostapchuk et al., 2006). Dynamics of IVa2 and the PD interactions were further characterized using purified IVa2 and 42–45 bp overlapping PD probes (Tyler et al., 2007). In agreement with the previous results, IVa2 demonstrated the highest affinity with a probe containing A1 and A2 repeats, followed by A2, A4-A5, A5-A7, and A3 repeats. Mutations in the CG motif, but not in the TTTG motif of the A1 repeat, disrupted IVa2 binding to the probe, reaffirming the earlier proposition that IVa2 nucleates the formation of protein complexes on the PD by binding to the CG motif. Additionally, purified IVa2 showed a very high affinity to the full-length PD producing several super-shifted complexes.

**Table 3:** **Composition of complexes detected by EMSA following virus infection or transient expression of IVa2 and L4 22K using a probe comprising A1 and A2 repeats (Ostapchuk et al., 2005, 2006)**.

**Complex**	**Components**	**Binding site**
1 (or y)	IVa2	CG motif of A1 repeat
2 (or x)	IVa2 + L4 22K	IVa2: CG motif of A1 repeat L4 22K: TTTG motif of A2 repeat
3	IVa2 + L4 22K	Complex 2 + additional IVa2: CG of A2 repeat

*Complex 1 and 2 are the same as complex y and x (Zhang and Imperiale, 2000), respectively*.

The 22K protein shares the N-terminal 105 amino acids with the L4 33K protein which plays an important role in capsid assembly and/or DNA packaging, thus raising the possibility that either 33K or 22K could be present in complex 2. The complexes 2 and 3 were detected by EMSA when nuclear extracts of cells expressing 22K and IVa2 were used with A1 and A2 repeats. Further support for the earlier finding that 22K is the unknown viral factor in complex x came from EMSA using purified 22K and IVa2 proteins and the packaging repeats (Ewing et al., 2007). In the absence of IVa2, purified 22K binds to the A1-A2 probe at very high concentration (>200 nM) and this binding is not affected by mutations in the TTTG motif, suggesting that the binding is non-specific. However, in the presence of IVa2 at its half-maximal binding concentration, 22K binds to the A1-A2 probe at very low concentrations (0.3–8 nM), resulting in formation of two complexes migrating slower than the complex detected when IVa2 alone binds to the A1-A2 probe. Further, as reported earlier, mutations in the TTTG or CG motifs abolished the slower migrating or all complexes, respectively. These experiments with the purified proteins confirmed that complex 1 contained IVa2 only, whereas complexes 2 and 3 contained IVa2 and 22K (Ewing et al., 2007). Later studies showed that the 33K protein also interacts with the PD (Ali et al., 2007; Ahi et al., 2015). 33K interacts with the A1 and A2 repeats (Ali et al., 2007) in the presence of IVa2 and this interaction results in the formation of two complexes similar to those reported earlier. In contrast, a study by Wu et al. (2013) showed that 33K does not preferentially bind to the PD. The reason for this discrepancy regarding the interaction of 33K with the PD is not clear. Given that 22K and 33K proteins share the N-terminal 100 amino acids, it is possible that both 22K and 33K interact with the packaging repeats in the presence of IVa2 and play an overlapping role.

#### IVa2

**Figure 4:**
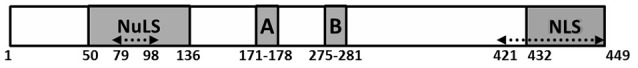
**Known functional domains of IVa2 protein**. Known functional domains of HAdV-C5 IVa2 protein (1–449 residues) are depicted. The locations of the nucleolar localization signal (NuLS), Walker A motif (A), Walker B motif (B), and nuclear localization signal (NLS) are shown. Dotted arrows (79–98, and 421–449) indicate the regions that are part of the bipartite DNA binding domain.

The AdV IVa2 gene is located between 11.3 and 16 map units of the HAdV-C5 genome. Transcription of the IVa2 gene occurs on the lower or anti-sense strand of the AdV genome from its own promotor (Binger and Flint, 1984; Winter and D'Halluin, 1991). IVa2 contains both a nuclear and nucleolar targeting signals (Figure 4). Consequently, the protein is targeted to the nucleolus in virus-infected cells (Lutz et al., 1996) and exists in an unprocessed form in the mature virions as well as in the light and heavy assembly intermediates (Winter and D'Halluin, 1991). IVa2 expression was detected as early as 12 h post-infection and continued to increase until 24 h post-infection. Expression was found to be dependent on cellular but not on viral DNA replication (Winter and D'Halluin, 1991; Lutz and Kedinger, 1996).

IVa2 is a multi-functional protein involved in various stages of the viral life cycle. Initial studies identified IVa2 as a transcriptional activator of the AdV major late promoter (MLP). IVa2 is a trans-acting factor and stimulates late gene transcription through its interaction with the downstream control region of MLP, which is composed of three regions, DE1, DE2a, and DE2b (Lutz and Kedinger, 1996). Striking homology between DE and PD was an impetus to examine the role of IVa2 in AdV DNA packaging (Lutz and Kedinger, 1996). Secondary structure analysis of IVa2 suggested that the DNA-binding domain of IVa2 consists of two parts, one closer to the N-terminus and the other toward the C-terminus, each contributing an amphipathic helix (Figure 4). The two amphipathic helices could assemble into a DNA-binding domain with the hydrophobic faces buried inside (Lutz and Kedinger, 1996). A recent study demonstrated the importance of the amphipathic helix at the C-terminus of IVa2 by deleting 10 amino acids. The chromatographic profile and a partial digestion with chymotrypsin and trypsin suggested that the deletion does not affect the tertiary structure of IVa2. However, the mutant failed to interact with the AdV PD, and therefore, could not support genome packaging (Christensen et al., 2012).

Initially, IVa2 was thought to play an important role in capsid assembly (Zhang and Imperiale, 2003). However, such a role was later ruled out since AdV mutants lacking IVa2 resulted in assembly of empty capsids, thereby reinforcing the role of IVa2 as a packaging ATPase (Ostapchuk et al., 2011). Several observations point toward the importance of IVa2 in AdV packaging. IVa2 interacts with the PD sequences, both *in vivo* (Zhang and Imperiale, 2000; Ostapchuk et al., 2005; Perez-Romero et al., 2005) and *in vitro* (Zhang and Imperiale, 2000; Tyler et al., 2007). Given the importance of the PD in genome packaging, it would not be surprising to find that IVa2 is an important packaging factor. IVa2 has been shown by immunogold labeling to be located at a single site on capsids (Christensen et al., 2008; Ahi et al., 2013). Copy number of a protein located at a single vertex is expected to be low. Based on the results of western blotting, metabolic labeling and quantitative mass spectrometry, the copy number of IVa2 was found to be six to eight molecules per virus particle (Christensen et al., 2008). The portal, one of the essential components of the genome packaging machinery, forms the central channel for passage of the genome during packaging, and is located at a unique vertex. The portal is essential for capsid assembly and is invariably present on the capsid as a dodecameric ring structure (Simpson et al., 2000; Orlova et al., 2003; Trus et al., 2004; Doan and Dokland, 2007). The presence of IVa2 on a unique vertex suggested that it may serve as the AdV portal. However, the low copy number of IVa2 on each virion and assembly of empty capsids in its absence suggested that IVa2 cannot function as a portal. If the AdV portal were to consist of only IVa2, the central pore would not be large enough for passage of the genome. It is possible that IVa2 in conjunction with another protein may constitute the portal; however, all portals known to date consist of a single protein. Therefore, we anticipate that the yet to be identified AdV portal will have key features similar to the known portals.

Studies with bacteriophages have shown that a motor protein is required to force the viral genome into a pre-formed capsid through the portal (Rao and Feiss, 2008). Most often, the motor protein is an ATPase, which utilizes the chemical energy of phosphodiester bonds to package the genome. Sequence analysis of AdV IVa2 revealed the presence of the Walker A and Walker B motifs, a characteristic feature of ATPases in the ABC and AAA+ families. (Figure 4). The Walker motifs in IVa2 are highly conserved among all AdVs, whereas the other parts of IVa2 are variable. Secondary structure predictions suggest that DNA packaging ATPases of phages and AdV IVa2 belong to the additional strand catalytic E (ASCE) class of ATPases although there are certain differences suggesting that they probably evolved independently (Burroughs et al., 2007). Changing a single residue from lysine to arginine or alanine within the Walker A motif was not tolerated, and thus, the mutant AdV genome was unable to generate infectious virus particles (Pardo-Mateos and Young, 2004). The lysine residue is critical for binding to ATP, and therefore, is important for the action of ATPases carrying the Walker motifs. Using purified IVa2 protein, it was shown that the integrity of Walker A and B motifs is important for ATP binding activity of IVa2 (Ostapchuk and Hearing, 2008). Additionally, HAdV-C5 genomes harboring IVa2 mutations in the ATPase domain failed to rescue an infectious virus, but instead resulted in accumulation of empty capsids, signifying that the block was at the DNA packaging step (Ostapchuk et al., 2011). It is conceivable that the ATPase function of an ATPase is activated only when this function is required. Therefore, the ATPase function of IVa2 would be initiated only during active genome packaging. Based on this hypothesis, we demonstrated that the ATPase function of IVa2 is activated in the presence of viral genome and L4 33K, another essential packaging factor (Ahi et al., 2015). This finding suggests that IVa2 serves as a motor protein for AdV DNA packaging, providing energy for pushing the genome into the capsid by ATP hydrolysis.

Apart from the full-length IVa2 which is approximately 50 kDa, a truncated isoform of roughly 40 kDa also exists in virus-infected cells, possibly due to translation initiation from methionine at position 75 (Pardo-Mateos and Young, 2004). In the absence of the full-length IVa2, the 40 kDa IVa2 produced two complexes in EMSA with the A1-A2 probe. Another study using a purified preparation of 40 kDa IVa2 showed that it has high affinity for binding to the A1-A2 repeats (Yang et al., 2009). Taken together, the ability of 40 kDa IVa2 to bind to the packaging sequences and the viability of mutant viruses expressing 40 kDa IVa2 indicates that the 40 kDa isoform is able to perform the packaging function.

#### L4 33K and 22K proteins

The AdV 33K protein is expressed from the L4 region of the major late transcription unit (MLTU). The predicted molecular weight of 33K is 24.9 kDa; however, the relative molecular weight was estimated to be around 39 kDa by SDS-PAGE (Oosterom-Dragon and Anderson, 1983). This discrepancy between the predicted and the observed molecular weight of 33K is mainly due to the high proline and glutamic acid residue content, hyper-phosphorylation and possible glycosylation of the 33K (Cauthen and Spindler, 1996). In infected cells, 33K localizes exclusively to the nucleus and organizes into unique granular structures (Gambke and Deppert, 1981).

Expression of 33K during the late phase suggested that it may play a role in upregulation of transcription from the MLTU and suppression of transcription from the early regions. Contrary to this hypothesis, a mutant virus (v33K.1) with a stop codon at position 20 of the 33K orf did not show any significant reduction in early or late gene expression or viral DNA replication, but had a 7-fold decrease in virus yield (Fessler and Young, 1999). The reduced virus yield was accounted for by a decrease in intermediate and mature virus particles. A stop codon at position 138 of the 33K orf did not significantly affect early or late gene expression, except that of proteins IIIa, and pVI (Wu et al., 2013). As observed by Fessler and Young, the 33K mutant described by Wu et al had a reduced yield of intermediate particles and completely fails to package the viral genome. The reduced yield of intermediate particles by 33K mutants despite lack of effect on late gene expression suggests a role of 33K in capsid assembly. In another study, deletion of 47 amino acid residues from the C-terminus of 33K completely abolished capsid assembly (Finnen et al., 2001). Thus, the studies by Fessler and Young, Wu et al and Finnen et al suggest a role of 33K in capsid assembly. However, the complete block in genome packaging in absence of 33K observed by Wu et al suggest that 33K is also important for genome packaging.

Evidence for a role of 33K in capsid assembly also comes from a study on bovine AdV serotype 3 (BAdV3). A homolog of the 33K gene is predicted in all AdV genomes (Vrati et al., 1995). Three distinct isoforms of the 33K protein, which may represent alternative splice variants, were detected in BAdV3-infected cells (Kulshreshtha et al., 2004). A stop codon at position 7 in the 33K orf resulted in a severe defect in genome packaging, but did not affect capsid assembly; however, a stop codon at position 97 resulted in complete loss of capsid assembly. These results are similar to those observed with HAdV-C5 33K mutants. A stop codon near the start of the orf may not prevent the expression of a smaller 33K isoform which may be sufficient for capsid assembly, but may not be fully active in performing its function in DNA packaging, thus resulting in accumulation of empty capsids and reduced mature virus yield. A stop codon further downstream in the 33K orf might block expression of all isoforms, thereby proving lethal due to a block in capsid assembly and implying a role of the C-terminal domain of 33K in capsid assembly. Collectively, these findings suggest that 33K has two functional domains acting at two different steps in AdV morphogenesis: a C-terminal domain functions during capsid assembly, and an N-terminal domain plays a role in DNA packaging. BAdV3 33K was shown to interact with the viral V and 100K proteins in virus-infected cells (Kulshreshtha and Tikoo, 2008). Interaction with 100K seems to be of significance with regard to hexon trimerization and its import to the nucleus on the basis of the following observations. In COS-7 cells, transiently expressed 100K failed to transport hexon into the nucleus, (Hong et al., 2005) and the majority of hexon remained in the cytoplasm of cells infected with a BAdV3 33K mutant (Kulshreshtha et al., 2004). These observations point toward a possible role of 33K in the nuclear transport of hexon. In insect cells, 100K alone was sufficient for hexon trimerization and its transport to the nucleus suggesting the presence of an unknown factor in the insect cells but not in COS-7 cells. Therefore, it is possible that 33K acts as a bridge between hexon trimerization and capsid assembly. Further experiments are warranted to explore this hypothesis.

Proteins that participate in genome packaging assemble at the “portal complex” which acts as a conduit for genome translocation during packaging. Thus, the constituting proteins of a portal complex assemble into ring-like oligomers. Purified HAdV-C5 33K has been demonstrated to form ring-like oligomers with a central channel (Ahi et al., 2015). In addition, 33K is located at a unique vertex on virus particles, interacts with IVa2 and stimulates its ATPase function (Ahi et al., 2013, 2015). These findings indicate that the function of 33K in genome packaging may be similar to that of the “small terminases” of bacteriophages (Roy et al., 2012; Sun et al., 2012).

22K is another AdV protein that plays a role in DNA packaging (Ostapchuk et al., 2006). In HAdV-C5, the orfs of 22K and 33K begin at nt 26195. The 22K orf continues uninterrupted until nt 26785. The 33K orf includes an intron between nts 26510 and 26713 and continues thereafter in a different reading frame than 22K until nt 27086. As a result, 22K and 33K share the N-terminal 105 amino acid residues, implying that they may have a shared function. 22K was identified as a constituent of the supershifted complexes using the packaging repeats as probes in gel shift assays, and this interaction was found to be dependent on the TTTG motif of the packaging repeats. Efficient *in vivo* DNA packaging correlated with the formation of these supershifted complexes on the packaging repeats, suggesting the importance of 22K in DNA packaging (Ostapchuk et al., 2005). Mutant AdV that does not express 22K assembles empty capsids, but they are defective in genome packaging (Wu et al., 2012). The genome packaging function of 22K is likely mediated by a conserved pair of cysteines and histidines in its C-terminal region (Guimet and Hearing, 2013).

#### L1 52/55K protein

The L1 52/55K protein plays an important role in AdV morphogenesis. The molecular weight of 48 kDa is predicted from its amino acid sequence, but it migrates on SDS-PAGE as a doublet at about 52 kDa. The two bands represent differentially phosphorylated forms of 52/55K. Under non-reducing conditions 52/55K forms higher molecular weight species suggesting it forms homo-oligomers, possibly involving disulfide linkages mediated by the Cys residue at position 24 (Hasson et al., 1989). 52/55K localizes to the nucleus, concentrating at the nuclear periphery at sites distinct from the viral DNA replication centers. HAdV-C5 mutants unable to express 52/55K or carrying a two amino acid substitution (EL334-335GP) fail to package DNA into empty capsids (Hasson et al., 1989; Gustin and Imperiale, 1998). The capsids assembled by these mutants were devoid of any viral DNA suggesting that 52/55K plays an important role in the recognition and/or incorporation of the viral DNA into empty capsids.

52/55K associates with the viral packaging sequences in virus-infected cells (Perez-Romero et al., 2005). The interaction seems to be mediated by another viral factor since the purified form of 52/55K did not bind to the packaging sequences (Ostapchuk et al., 2005; Perez-Romero et al., 2005). Interestingly, IVa2 interacts with 52/55K suggesting that 52/55K may bind to the PD in the presence of IVa2 (Gustin et al., 1996). However, the interaction of IVa2 and 52/55K with the PD was shown to be independent of each other (Perez-Romero et al., 2005). Interaction of 52/55K with IVa2 is mediated by the N-terminal 173 amino acids, whereas the genome packaging function lies in the N-terminal 331 amino acids. Interestingly, the N-terminal 331 amino acids are also essential for interaction with the PD *in vivo*, implying that 52/55K interaction with the packaging sequences is essential for its function in genome packaging (Perez-Romero et al., 2005). Empty capsids assembled by IVa2-null HAdV-C5 mutants contain 52/55K in amounts comparable to wild type virus, indicating that interaction with IVa2 is not necessary to recruit 52/55K to empty capsids (Ostapchuk et al., 2011). 52/55K also interacts with AdV IIIa, and this interaction contributes to serotype-specific DNA packaging (Ma and Hearing, 2011). Interestingly, 52/55K also binds to the major core protein pVII (which also binds to IVa2) and to the viral genome in a non-specific manner. The importance of interactions of pVII with 52/55K or IVa2 is not clear; however, given that pVII binds to unpackaged genome, its interactions with the packaging machinery will likely play a crucial role during genome packaging (Zhang and Arcos, 2005).

Interestingly, a consensus recognition cleavage site for the AdV 23K protease (AVP) is present in 52/55K (Webster et al., 1989), and this site is conserved among various AdV serotypes (Mangel and San Martin, 2014). A recent study demonstrated that 52/55K is indeed a substrate for AVP (Perez-Berna et al., 2014). 52/55K seems to be in an extensive network of interactions with capsid proteins including hexon and penton (shell), and pVIII and VII, in addition to interactions with IIIa and IVa2 mentioned above. 52/55K is cleaved by AVP, presumably concomitant with genome packaging, at a canonical and multiple non-canonical consensus sequences into fragments corresponding to molecular weights of 40, 34, 30, 27, and 18 kDa, with 40 kDa being the predominant fragment. The cleavage of 52/55K by AVP, similar to its other substrates, is DNA-dependent, and results in the loss of interaction of 52/55K with other capsid proteins and the viral genome (Perez-Berna et al., 2014). Since none of the fragments of 52/55K appear in the mature virus, they are presumably ejected during or after packaging by an unknown mechanism. Condezo et al. (2015) described two light assembly intermediates, L2 and L3, of HAdV-C5 mutants with delayed packaging. The maturation state of 52/55K was found to be different in L2 and L3. L2 contains a larger fraction of full-length 52/55K than L3, whereas in L3, the fragments are more abundant. In L2, 52/55K is located along the vertices, possibly in close association with IVa2 at the portal vertex and with IIIa at other vertices (Condezo et al., 2015). Given that 52/55K can interact with the incoming genome due to its non-specific affinity to DNA, it can be hypothesized that 52K/55K retains the incoming genome inside the capsid shell, thus playing an important role in organizing the packaged genome inside the shell. The observation that L3 but not L2 is associated with the genome suggests that the cleavage of 52/55K by AVP is a dynamic process that occurs during or after genome packaging.

#### E2 72K protein

E2 72K, also known as DNA-binding protein (DBP), interacts with the packaging proteins IVa2 and 33K and is located at a unique vertex of AdV (Christensen et al., 2008; Ahi et al., 2013). Although the majority of studies on DBP have focused primarily on its role in viral DNA replication, the studies from our laboratory (Christensen et al., 2008; Ahi et al., 2013) and another report (Nicolas et al., 1983) suggest that it may play an important role in capsid assembly and/or genome packaging.

The DBP is expressed from the early region 2A (E2A) in large amounts in virus-infected cells. It is essential for viral DNA replication, although it also plays important roles in determination of the host range, stability of viral mRNAs, and host-cell transformation (Rice et al., 1987; Cleghon et al., 1989; Harfst and Leppard, 1999). HAdV-C5 DBP contains two distinct domains, 44K and 26K. The C-terminal 44K domain contains amino acid residues from 174 to 529 and is sufficient for DNA binding and *in vitro* DNA replication (Ariga et al., 1980). This globular 44K fragment has a 17-amino acid residue extension at the C-terminus that is essential for multimerization of DBP on the displaced ssDNA during DNA replication (Tucker et al., 1994; Dekker et al., 1997). The 26K N-terminal domain contains several phosphorylation sites. Mutations affecting the C-terminal domain block the role of DBP in DNA replication, but do not affect its gene expression activity (Klein et al., 1979). Two distinct classes of DBP can be recognized in virus-infected cells by cell fractionation and immunofluorescence. The first subclass is organized as globular structures inside the nucleus and is primarily involved in active DNA replication. The second subclass is more diffused within the nucleus (Voelkerding and Klessig, 1986).

Interestingly, a HAdV-C5 mutant with point mutations affecting DBP is temperature-sensitive for capsid assembly in HEK293 cells, but not in HeLa cells, suggesting that DBP plays an essential role in virus assembly through interaction with some host-specific factors. Importantly, at non-permissive temperature, the mutant virus synthesizes the late viral structural proteins in amounts similar to the wild-type virus, but does not assemble capsids (Nicolas et al., 1983). Since AdV capsid assembly and DNA replication occurs in distinct compartments of the nucleus (Hasson et al., 1992), the diffusely staining subclass of DBP may participate in capsid assembly. Currently, the role of DBP in AdV capsid assembly or genome packaging is not clearly understood and awaits further exploration.

### Cellular proteins

Nuclear extracts from AdV-infected or uninfected cells were incubated with a probe consisting of the A5-7 repeats of the AdV PD and analyzed initially by EMSA (Schmid and Hearing, 1998). This led to identification of two cellular proteins: P-complex and COUP-TF. The binding of P-complex to various A-repeats correlated with the packaging efficiency of the mutants containing multimeric copies of different A-repeats. In contrast, the binding of COUP-TF was strongest to the A-repeat with weakest packaging efficiency. The findings suggested that P-complex, but not COUP-TF, may be important for AdV genome packaging (Erturk et al., 2003). This study also showed that a third cellular protein, Oct-1, binds to the PD, but does not seem to play a functional role in the packaging process as its binding ability to the A-repeats does not correlate with the packaging efficiency. P-complex contains a full-length CDP, which was identified initially as a cell cycle control factor (Bodmer et al., 1987; Moon et al., 2000). Interestingly, the full-length CDP was also found in purified virus particles. A mutation in the TTTG motif of a critical A-repeat within the PD completely abolished genome packaging without any effect on the binding of CDP (Ostapchuk et al., 2005). These observations suggest that CDP binding is not required for genome packaging.

## Serotype specificity of AdV DNA packaging

Alignment of the PDs of AdV serotypes belonging to different species revealed the conservation of bipartite consensus motifs of packaging repeats implying that the packaging of AdV genomes into non-homologous capsids may be possible (Ostapchuk and Hearing, 2001). Results of independent studies have indicated that AdV DNA packaging seems to be serotype-specific (Zhang et al., 2001; Wohl and Hearing, 2008; Ma and Hearing, 2011). IVa2 and 22K proteins, but not L1 52/55K of HAdV17 serotype are capable of complementing function of the respective HAdV-C5 proteins, suggesting that 52/55K is important for serotype specificity of AdV DNA packaging (Wohl and Hearing, 2008). Similarly, an earlier study reported that co-infection with HAdV7, HAdV12 and HAdV17 cannot complement the growth of a HAdV-C5 52/55K-null mutant (Zhang et al., 2001). It seems that the N-terminal 191 amino acids are essential for imposing serotype-specific function of 52/55K (Wohl and Hearing, 2008). Since 52/55K does not bind to the A-repeat consensus independently or in the presence of IVa2, it is possible that its role in serotype specificity of genome packaging is mediated by another protein. Interestingly, 52/55K interacts with the structural protein IIIa from the same AdV serotype and this interaction is essential for DNA packaging (Ma and Hearing, 2011) suggesting that IIIa and 52/55K act together and their serotype-specific interaction is the basis for serotype-specific DNA packaging. Based on the observation that IIIa binds to the PD *in vivo*, it is possible that IIIa is the previously unknown factor that recruits 52/55K to the PD (Ma and Hearing, 2011).

Pre-existing immunity is one of the limitations of AdV-based vectors in gene delivery applications. Since hexon is the dominant antigenic determinant of AdV capsids, it is plausible that packaging of the genome of one serotype into the capsids of another serotype might help to overcome the pre-existing immunity hurdle. Co-infection of the HAdV-C5 temperature-sensitive mutant (Ad5ts147) carrying mutations in the hexon gene with either HAdV2, HAdV3, HAdV4 or HAdV9 resulted in “pseudopackaging” of the Ad5ts147 genome into the capsids of the heterologous serotype and the generation of infectious virus particles (Ostapchuk and Hearing, 2001). This approach provides an interesting alternative for circumventing pre-existing immunity. It will be necessary to block the packaging of the wild-type genome. A straightforward way may be to flank the PD of the wild-type genome with *loxP* sites so that the PD can be excised by Cre recombinase.

## Evidence for sequential assembly of AdV

### Empty capsids and intermediates in AdV assembly

In viruses that follow the sequential assembly pathways, the empty capsids, also known as procapsids or proheads, are essential assembly intermediates. Three components that are essential for capsid assembly include the coat (major capsid), the scaffolding and the portal proteins. If AdV follows the sequential assembly pathway, it must first assemble the empty capsids before genome packaging, which means that the empty capsids must be detected during virus infection. Indeed, empty capsids (1.29 g/cm^3^) can be readily detected in virus-infected cells (Ishibashi and Maizel, 1974; Edvardsson et al., 1976; Ostapchuk et al., 2011). Pulse-chase labeling kinetics demonstrated that the empty capsids are precursors to the mature virions (Sundquist et al., 1973; Ishibashi and Maizel, 1974). In addition to the empty capsids that lack any DNA, two classes of assembly intermediates, called light and heavy, exist in virus-infected cells. The light assembly intermediates are associated with DNA fragments derived from the left end of viral genome (Daniell, 1976; Khittoo and Weber, 1981). Interestingly, the light intermediates range in density from 1.29 g/cm^3^ (empty capsids) to 1.34 g/cm^3^ (mature virions), with longer DNA fragments associated with the particles of higher densities (Tibbetts, 1977). The light intermediate particles possibly represent capsids in which packaging was initiated but could not be completed, presumably due to limiting packaging factors or simply represent capsids that were in the process of packaging at the time of harvesting of the cells and the genome was fragmented during virus purification. The heavy assembly intermediates (1.37 g/cm^3^) contain DNA with a sedimentation coefficient similar to that of the viral genome in the mature virus (D'Halluin et al., 1978a), suggesting that they represent particles after packaging. Pulse-chase labeling kinetics demonstrated that light particles are precursors to heavy particles, which themselves are precursors to the mature viruses (Weber, 1976; D'Halluin et al., 1978a,b; Edvardsson et al., 1978), thus suggesting that AdVs follow the sequential assembly pathway. Khittoo and Weber (1977) analyzed a temperature sensitive (ts) mutant of HAdV-C2 (ts4) that accumulates light particles at non-permissive temperature. Interestingly, the light particles assembled by ts4 mutant at non-permissive temperature failed to convert to mature viruses even after shift-down to permissive temperature. Similarly, a ts mutant of HAdV-C5 with a mutation in 52/55K assembled light particles at non-permissive temperature that failed to convert to mature viruses at permissive temperature (Hasson et al., 1989). These findings suggest that the light particles are not bona fide assembly intermediates, but represent dead-end products. However, it must be considered that these studies were done with mutant AdVs. It is possible that once encapsidation is blocked at non-permissive temperature, it does not restart even after shift-down to the permissive temperature. In addition, the ts4 mutant of HAdV-C2 (Khittoo and Weber, 1977) accumulates light particles in greater amounts than the wild type virus even at permissive temperature, suggesting that the mutant is not fully functional at permissive temperature. Several recent reports have shown that AdVs lacking any one of the functional packaging proteins (IVa2, 33K or 22K) fail to package the genome, resulting in accumulation of empty capsids in the mutant virus-infected cells (Ostapchuk et al., 2011; Wu et al., 2012, 2013; Guimet and Hearing, 2013). Furthermore, mutation affecting a single residue in the P-loop motif of IVa2, the packaging ATPase, also results in accumulation of empty capsids. Thus, either the absence of any one packaging factor, or a block in the ATPase function of the packaging ATPase results in accumulation of empty capsids. Further evidence for the sequential assembly pathway comes from the helper-dependent (HD) AdV vector system (Parks et al., 1996). In this system, removal of the PD of a helper virus is facilitated by *cre*-mediated excision. Deletion of the PD from the helper virus genome results in the accumulation of empty capsids (Stilwell et al., 2003; Ahi et al., 2013). This observation indicates that the empty capsids are intermediates in the packaging process.

### Scaffolding proteins

Scaffolding protein-assisted oligomerization of the coat protein is also seen in AdVs. A characteristic feature of a scaffolding protein is that it exits the empty capsid and is degraded after packaging implying its presence in the empty capsids but not in the mature virions (Dokland, 1999). The 52/55K protein is present in assembly intermediates, but not in mature virions (Hasson et al., 1992), suggesting that it might act as a scaffolding protein. However, empty capsids can be assembled in absence of the 52/55K, proving that it does not act as a scaffolding protein (Gustin and Imperiale, 1998). During the late phase of AdV infection, L4 100K modulates cellular expression to promote translation of AdV late mRNAs (Hayes et al., 1990). 100K also acts as a chaperone-like protein and thereby promotes trimerization of hexon followed by its transport to the nucleus (Cepko and Sharp, 1982). In addition to these functions, several observations indicated that 100K may serve as the scaffolding protein for AdV capsid assembly (Morin and Boulanger, 1984, 1986). 100K accumulates in the nucleus during the late stage in virus infection and is present in the early assembly intermediate particles. There was a lack of capsid assembly by a temperature-sensitive 100K mutant (H2ts107), but it was capable of hexon trimerization and its nuclear transport at the restrictive temperature. It appears that hexon trimerization and its nuclear transport functions are mediated by distinct domains of 100K. Interestingly, the scaffolding function of 100K seems to be localized in its central domain between amino acids 300–400 (Morin and Boulanger, 1984).

The aforementioned observations only provide a limited insight into the scaffolding function of AdV. The following questions are still unanswered: Where and how does the capsid assembly occur? Does a portal or a similar structure serve as a nucleation point for capsid assembly? How many copies of L4 100K are present in the assembly intermediates or what is the optimal ratio of hexon trimer to 100K for efficient assembly? How and when does 100K exit the capsids after assembly, or is it degraded by AdV protease or by another mechanism? Cleavage by viral protease is required for the transport of BAdV3 100K protein to the nucleus, but a similar observation is not reported for other AdVs (Makadiya et al., 2015). Interestingly, 100K of HAdV-C5 is cleaved by a host cell-encoded serine protease and GranzymeB into 70, 80 and 95 kDa fragments (Andrade et al., 2007). A 90 kDa cleavage product of 100K is also detected in vast amounts in H2ts107 assembly intermediates (Morin and Boulanger, 1986); however, it is not yet clear if these cleavage products are related. The 90kDa species detected in H2ts107 assembly intermediates could have a differential role in assembly than the full-length 100K since 90kDa forms a complex with the groups of nine hexons, whereas the full-length 100K seems to be associated with IIIa and penton base located at vertices (Morin and Boulanger, 1986).

### DNA encapsidating motor complex

In viruses known to use the sequential packaging pathway, the portal serves as a site for assembly of the DNA packaging motor. The minimal, essential components of the packaging motor in known viruses include an enzyme with ATPase activity, known as “large terminase” or “packaging ATPase,” and a protein that helps in DNA recognition and potentiates ATPase activity, known as the “small terminase.” The ATPase activity is essential for pushing the DNA into a relatively small volume inside the capsid (Chelikani et al., 2014). The function of IVa2 in AdV packaging is presumably comparable to that of the packaging ATPases. We have demonstrated that purified L4 33K protein stimulates the ATPase activity of IVa2 (Ahi et al., 2015). This result suggests that 33K is equivalent to a small terminase protein; however further characterization of this function of 33K is necessary. It is possible that the genome packaging step in AdVs involves more components than those mentioned above. Also, both 22K and 33K seem to be essential for the genome packaging step, and the absence of either one results in accumulation of empty capsids (Wu et al., 2012, 2013; Guimet and Hearing, 2013). Therefore, it is possible that both proteins function cooperatively.

### Similarities between AdV and phage PRD1

Remarkable structural similarities between AdVs and bacteriophage PRD1 have been observed (Krupovic and Bamford, 2008). AdVs and PRD1 share the following features: a linear dsDNA genome with ITRs of similar size and a covalently attached terminal protein; protein-primed replication of the viral genome; capsids with a pseudo *T* = 25 lattice; trimeric major capsid proteins (MCP) with a double beta barrel fold that gives rise to a pseudohexagonal arrangement of the MCP (Benson et al., 2004); vertices occupied by pentameric proteins that are associated with a spike protein or a fiber; and the presence of a virion-associated packaging ATPase-like protein (Stromsten et al., 2005; Huiskonen et al., 2007).

The PRD1 genome is packaged into preformed capsids through a DNA packaging complex located at a unique vertex (Hong et al., 2014). Because of the striking parallels between AdVs and PRD1, it is likely that the genome packaging in AdV follows a pathway similar to PRD1. Genome packaging in PRD1 is assisted by the virion-associated packaging ATPase, protein P9 (Stromsten et al., 2005). It is interesting to note that IVa2, the AdV equivalent of the packaging ATPases, is also a virion-associated protein. Four virus-encoded proteins, P6, P9, P20 and P22, constitute the unique vertex of PRD1 (Hong et al., 2014). One notable feature of PRD1 is the presence of an internal membrane. The proteins P20 and P22 constitute the transmembrane conduit for the passage of the genome during packaging, whereas P6 acts as a packaging efficiency factor, which assists the packaging ATPase P9 (Stromsten et al., 2005; Hong et al., 2014). Although existence of a unique vertex in AdV has been reported, the components of the AdV unique vertex have not been fully determined; however, the presence of IVa2, 33K and DBP on a single location (Ahi et al., 2013) on the viral capsid suggest that they likely constitute the AdV unique vertex. Although AdV shares several features with PRD1, they do not have a similar membrane component as seen in PRD1, suggesting that the mechanistic details of the AdV packaging process may differ from that of PRD1.

## Concluding remarks

Extensive characterization of the cis-acting PD sequences of the AdV genome has led to the identification of several trans-acting factors that bind to the PD and are important for genome packaging. Localization of IVa2, 33K and DBP on a unique vertex provided strong evidence that the AdV likely harbors a structure equivalent to the portal complex observed in dsDNA bacteriophages and herpesviruses. Additionally, the accumulation of empty capsids in the absence of a functional IVa2, 22K, 33K or 52/55K strongly suggest that AdV follows a sequential assembly pathway. It is not clear how and when each of these proteins participates during genome packaging, and thus further exploration is necessary to precisely dissect their roles. Taking into consideration the advancement in the understanding of capsid assembly and genome packaging in dsDNA bacteriophages, the knowledge of AdV assembly and packaging is still in its infancy. AdV being a mammalian virus, it will not be surprising if its packaging system differs from that observed in bacteriophages. Characterization of the AdV portal by virological, biochemical and structural studies will be critical for defining the structural and functional aspects of the AdV packaging motor complex. Study of each component of the AdV packaging process by advanced structural biology techniques (such as cryo-electron tomography and atomic force microscopy) will illustrate their structural features and the domains that are involved in homo-oligomerization and that participate in interactions with other components of the packaging complex. Understanding of AdV packaging process will elucidate the necessary components and conditions for developing an *in vitro* packaging system for AdV, which will help in generating next generation of AdV vectors with better cell targeting and vaccine immunogenicity, but reduced vector immunity.

## Author contributions

YA and SM wrote the manuscript.

### Conflict of interest statement

The authors declare that the research was conducted in the absence of any commercial or financial relationships that could be construed as a potential conflict of interest.
